# Aptamer-Based Electrochemical Biosensors for the Detection of *Salmonella:* A Scoping Review

**DOI:** 10.3390/diagnostics12123186

**Published:** 2022-12-16

**Authors:** Nor Syafirah Zambry, Mohamad Ahmad Najib, Mohd Syafiq Awang, Kasturi Selvam, Muhammad Fazli Khalid, Yazmin Bustami, Hairul Hisham Hamzah, Mehmet Ozsoz, Asrulnizam Abd Manaf, Ismail Aziah

**Affiliations:** 1 Institute for Research in Molecular Medicine (INFORMM), Health Campus, Universiti Sains Malaysia, Kubang Kerian 16150, Kelantan, Malaysia; 2 Collaborative Microelectronic Design Excellence Centre (CEDEC), Sains@USM, Level 1, Block C, No. 10 Persiaran Bukit Jambul, Bayan Lepas 11900, Pulau Pinang, Malaysia; 3 School of Biological Sciences, Universiti Sains Malaysia, George Town 11800, Pulau Pinang, Malaysia; 4 School of Chemical Sciences, Universiti Sains Malaysia, George Town 11800, Pulau Pinang, Malaysia; 5 Department of Biomedical Engineering, Near East University, Nicosia 99138, Turkey

**Keywords:** *Salmonella*, foodborne disease, electrochemical aptasensor, detection, scoping review

## Abstract

The development of rapid, accurate, and efficient detection methods for *Salmonella* can significantly control the outbreak of salmonellosis that threatens global public health. Despite the high sensitivity and specificity of the microbiological, nucleic-acid, and immunological-based methods, they are impractical for detecting samples outside of the laboratory due to the requirement for skilled individuals and sophisticated bench-top equipment. Ideally, an electrochemical biosensor could overcome the limitations of these detection methods since it offers simplicity for the detection process, on-site quantitative analysis, rapid detection time, high sensitivity, and portability. The present scoping review aims to assess the current trends in electrochemical aptasensors to detect and quantify *Salmonella*. This review was conducted according to the latest Preferred Reporting Items for Systematic review and Meta-Analyses extension for Scoping Reviews (PRISMA-ScR) guidelines. A literature search was performed using aptamer and *Salmonella* keywords in three databases: PubMed, Scopus, and Springer. Studies on electrochemical aptasensors for detecting *Salmonella* published between January 2014 and January 2022 were retrieved. Of the 787 studies recorded in the search, 29 studies were screened for eligibility, and 15 studies that met the inclusion criteria were retrieved for this review. Information on the *Salmonella* serovars, targets, samples, sensor specification, platform technologies for fabrication, electrochemical detection methods, limit of detection (LoD), and detection time was discussed to evaluate the effectiveness and limitations of the developed electrochemical aptasensor platform for the detection of *Salmonella*. The reported electrochemical aptasensors were mainly developed to detect *Salmonella enterica* Typhimurium in chicken meat samples. Most of the developed electrochemical aptasensors were fabricated using conventional electrodes (13 studies) rather than screen-printed electrodes (SPEs) (two studies). The developed aptasensors showed LoD ranges from 550 CFU/mL to as low as 1 CFU/mL within 5 min to 240 min of detection time. The promising detection performance of the electrochemical aptasensor highlights its potential as an excellent alternative to the existing detection methods. Nonetheless, more research is required to determine the sensitivity and specificity of the electrochemical sensing platform for *Salmonella* detection, particularly in human clinical samples, to enable their future use in clinical practice.

## 1. Introduction

*Salmonella* is a significant pathogenic bacteria that causes major foodborne disease in humans and animals, called salmonellosis [1,2,3]. The *Salmonella* genus is classified into two distinct species, namely *Salmonella enterica* and *Salmonella bongori*. *Salmonella enterica* has been recognized as the predominant species responsible for foodborne disease outbreaks in numerous countries, resulting in thousands of deaths globally [2,4]. More than 2600 serovars of *Salmonella enterica* have been reported in foodborne outbreaks [5]. Among these serovars, *Salmonella enterica* serovar Typhimurium and *Salmonella enterica* serovar Enteritidis were the main isolated serovars responsible for human disease across countries [3,4]. *Salmonella* infection can be acquired through contaminated foods regularly found in meat products, eggs, dairy products, vegetables, and water [6].

This pathogen infects humans and animals through the intestinal tract. It may result in mild clinical symptoms such as abdominal pain, vomiting, headache, fever, self-limiting diarrhea to severe dehydration, and fever (typhoid) that can cause death without immediate medication [7,8,9]. The severity of *Salmonella* infection depends on the serovar and health status of the infected individual. The infection mainly occurs in vulnerable communities, including children under five years old, the elderly, and immunosuppressed individuals [10,11].

The spread of *Salmonella* infection can be significantly controlled by the emergence of reliable and effective detection methods for monitoring *Salmonella* contamination in the food chain (food handling, preparation, and storage) [12,13]. The standardized conventional microbiological method, according to the International Organisation of Standards (ISO) (ISO 6579:2002), has been used as the gold standard for monitoring *Salmonella* contamination in foods (10^2^–10^3^ CFU/mL) [6,14,15,16,17]. The method involves a pre-enrichment step of the samples in buffered peptone water followed by enrichment in selective media (e.g., Rappaport Vassialidis soy (RVS) broth and Muller Kauffmann tetrathionate-novobiocin). The enriched samples are then subjected to culturing on differential media (e.g., Xylose Lysine Deoxycholate (XLD) and Hoektoen) using a plating method. The suspected colonies are further confirmed using biochemical and serological tests [17,18]. The above-mentioned process is laborious and time-consuming as the results can only be confirmed within two to seven days, which is difficult for testing a large number of samples [2,16,19]. Moreover, the microbiological method is challenging due to the viable but non-culturable (VBNC) state of pathogens [20].

The advancement in *Salmonella* detection methods allows the emergence of rapid nucleic-acid-based assays and immunological-based assays that are routinely used in diagnostic laboratories [21]. In a nucleic-acid-based assay, *Salmonella* is detected via amplification of the specific fragments with or without hybridization of a probe. Polymerase chain reaction (PCR) and real-time PCR are being used for qualitative and quantitative diagnostics, respectively, based on their high accuracy, reliability, high sensitivity, and specificity (the turnaround time from the sample preparation to the detection process is approximately 16 h) [19,22]. Aside from accelerating the *Salmonella* detection time, this method exhibits comparable sensitivity and specificity to the conventional method. Notably, the PCR-based assay can detect *Salmonella* concentration down to 10^4^ CFU/mL after the enrichment procedure [16].

Enzyme-linked immunosorbent assay (ELISA) is the most prevalent immunological-based assay for the detection of *Salmonella* [19]. Similar to PCR-based assays, ELISA can give a sensitivity of 10^4^−10^5^ CFU/mL, making it comparable to the conventional microbial method [2]. Many ELISA test kits are available on the market due to their ability for high-throughput food testing [23]. The BIOLINE *Salmonella* ELISA Test is one of the available commercial ELISA test kits to detect *Salmonella* in food products with a turnaround time of approximately 36 h, including the enrichment step [24]. Although the existing *Samonella* detection methods are fast, reliable, and sensitive, they are not preferable for on-site detection due to the requirement for sophisticated instruments and trained personnel [25]. Due to these limitations, the focus on developing a simple, practical, fast, inexpensive, and sensitive detection method has been conducted to reduce the incidence of outbreaks that threaten global public health.

More studies have been performed on electrochemical biosensors to detect various pathogens due to their ability to provide accurate and sensitive detection, fast response time, automation, affordability, ease of operation, miniaturization ability, and portability. These promising characteristics make electrochemical biosensors highly preferable for on-site and real-time pathogen testing in food samples. An electrochemical biosensor for pathogen detection converts the biochemical reactions that occurr between the immobilized biorecognition molecules (e.g., antibodies, enzymes, aptamers, DNA, or antimicrobial peptides) and target pathogens on the surface of conducting material, known as the working electrode, into measurable electrical signals (current, impedance, potential, or conductance). The electrical signals quantitatively represent the concentration of pathogens in the samples [26,27,28].

Aptamer is a short single-stranded oligonucleotide commonly synthesized using the Systematic Evolution of Ligands by Exponential Enrichment (SELEX) technique [29]. It is used as one of the biorecognition molecules in electrochemical sensing platforms due to its advantages of stability at a wide range of temperatures, ability to detect various types of target (e.g., drugs, proteins, antibiotics, cells), robust affinity, and specificity for their target molecules [30,31]. Due to their excellent characteristics and ease of synthesis, aptamer-based electrochemical sensing platforms known as electrochemical aptasensors are often chosen in many diagnostic fields [32]. Curti et al. [33] have proven the efficiency of electrochemical aptasensors in detecting the contagious virus SARS-CoV-2. In another study, Park et al. [34] demonstrated the development of an electrochemical aptasensor for Zika virus detection in human serum samples. However, limited studies are available on developing electrochemical aptasensors for detecting bacterial pathogens, especially the genus *Salmonella*. According to Subjakova et al. [35], only 46 research articles have been published on the electrochemical bacterial aptasensor. Therefore, this review aims to provide an overview of the current trends in the development of electrochemical aptasensors for *Salmonella* detection. Several important criteria, such as the *Salmonella* serovars, sensor specification, platform strategies, electrochemical detection method, limit of detection (LoD), and detection time have been discussed to evaluate the effectiveness and limitations of the existing electrochemical aptasensors for *Salmonella* detection.

## 2. Methods

The present study was performed as a scoping review to provide a concise overview of the currently available studies on electrochemical aptasensors for *Salmonella* detection [36,37]. The review adopted the latest Preferred Reporting Items for Systematic review and Meta-Analyses extension for Scoping Reviews (PRISMA-ScR) guidelines [36,38].

### 2.1. Search Strategy 

A comprehensive search was conducted using the specified keywords in three databases (PubMed, Scopus, and Springer) in January 2022. The keywords used for the search are “aptamer” and “*Salmonella*”. These keywords were combined with the Boolean operators AND producing the search string “(aptamer) AND (*Salmonella*)”. The search string was used for all three databases without any filters. Furthermore, a list of references from the retrieved literature was manually screened as an additional search in the protocol.

### 2.2. Selection of Studies

Articles were excluded if (i) the studies were not relevant to the development of electrochemical aptasensors for the detection of *Salmonella*; (ii) the studies were published in languages other than English or Malay; (iii) the studies were reported in the form of book chapters, encyclopedia articles, conference proceedings, mini-reviews, systematic reviews, or review articles. Only research articles were selected in this scoping review. The search results were downloaded into Mendeley, and duplicates were filtered and removed. The collected research titles and abstracts were screened independently by five authors (N.S.Z., M.AN., K.S., M.S.A., and M.F.K.) by referring to the selection criteria. A satisfactory agreement for the screening process was assessed between the authors. Abstracts that fulfilled the selection criteria were then subjected to full-text screening. Discrepancies during full-text screening were resolved through discussion among all authors. The selected full texts were evaluated by all authors to summarize the findings.

### 2.3. Data Extraction

Details of the included studies were extracted and summarized in a table. The following data were extracted: *Salmonella* serovars, targets, samples, sensor specification, platform technologies for fabrication, detection methods, LoD, and detection time. Specifically, descriptive analyses were performed by serovars, samples, sensor specification, and detection methods.

## 3. Results

### 3.1. Search Results

**Figure 1:**
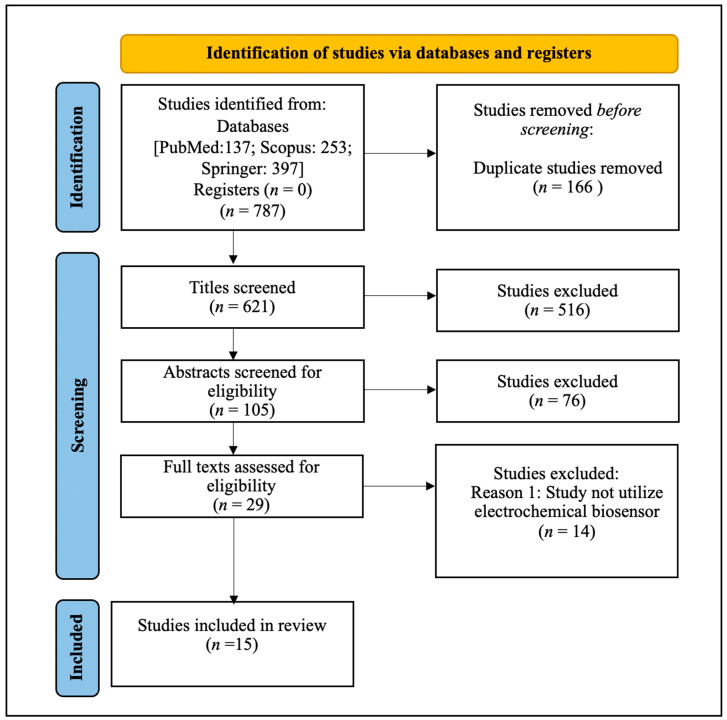
PRISMA-ScR flowchart of the scoping review process.

A total of 787 studies was identified in three databases (Figure 1). Of these, 166 duplicates were removed, resulting in 621 studies being subjected to title screening. After screening the titles, a total of 516 studies that were unrelated to *Salmonella* spp. or electrochemical biosensors were excluded. The remaining 105 studies were screened for abstract eligibility, and 76 irrelevant studies were excluded. Finally, 29 studies were appraised for eligibility, and 14 that were related to *Salmonella* spp. but did not involve electrochemical biosensors were excluded. A total of 15 studies was included in the final review. The collated studies demonstrate the publications of the development of electrochemical aptasensors targeting *Salmonella* from January 2014–January 2021. Table 1 summarizes the key characteristics of the included studies, namely *Salmonella* serovars, targets, samples, sensor specification, platform technologies for fabrication, detection methods, LoD, and detection time.

### 3.2. Salmonella Serotype, Target, and Sample Matrix

**Figure 2:**
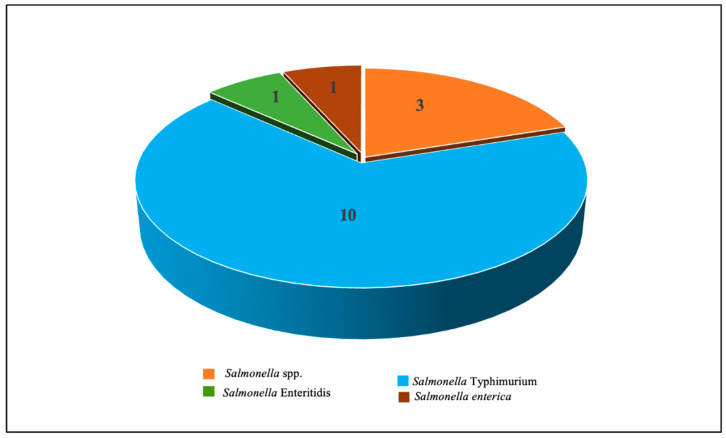
*Salmonella* serovars involved in the development of electrochemical aptasensors in 15 studies.

Figure 2 demonstrates the *Salmonella* serovars commonly studied for the development of electrochemical aptasensors. Of the 15 studies retained for review, ten studies of electrochemical aptasensors were developed for detecting *S.* Typhimurium, followed by three studies for detecting *Salmonella* spp. and one study for detecting both *S.* Enteritidis and *S. enterica*. This finding demonstrates that most developed electrochemical aptasensors detect *S*. Typhimurium rather than other *Salmonella* serovars. All studies detect the whole cell of *Salmonella* as a target in various types of food samples, namely chicken meat (six studies), milk (four studies), apple juice (two studies), egg (two studies), and pork (one study) (Table 2). Among these food samples, most studies reported *Salmonella* in chicken meat due to the high contamination of this pathogen in this type of sample [54].

### 3.3. Sensor Specification and Platform Technology for Fabrication

**Figure 3:**
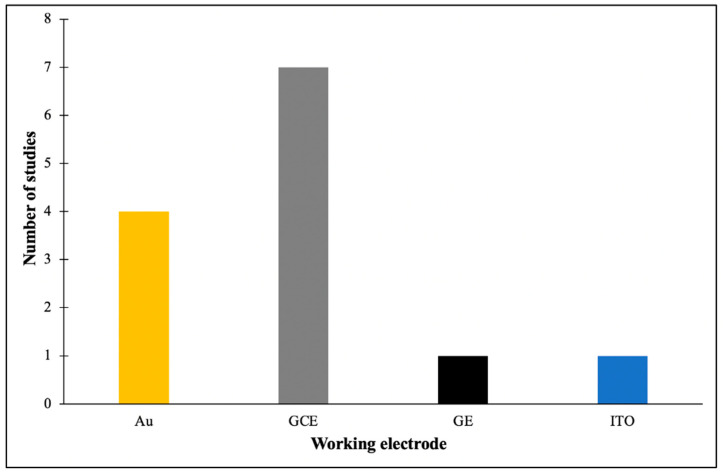
The type of working electrode employed in the fabrication of electrochemical aptasensors for detection of *Salmonella.* Au = Gold; GCE = Glassy carbon electrode; GE = graphite electrode; ITO = indium tin oxide.

The sensor specification often determines the detection performance (LoD and detection time) of the electrochemical aptasensor platform. Various sensing strategies have been employed in the fabrication of electrochemical aptasensors to detect *Salmonella*. As shown in Figure 3, most of the electrochemical aptasensors were fabricated using a glassy carbon electrode (GCE) (seven studies) and gold (Au) (four studies) as the working electrode. Only one study employed indium tin oxide (ITO) and graphite electrodes in the fabrication of an electrochemical aptasensor for detecting *Salmonella*. The study revealed that the working electrodes were mainly modified with nanomaterials such as gold nanoparticles (AuNPs) (six studies), reduced graphene oxide (rGO)(four studies), multi-walled carbon nanotubes (MWCNTs) (two studies), graphene (one study), graphene oxide (GO) (one study), and nanoporous gold (NPG) (one study) to give the most sensitive electrochemical detection of *Salmonella* (Table 3). The advanced electrochemical biosensor technology allows miniaturization of electrodes, such as screen-printed electrodes (SPEs) [55]. Although most sensor-related studies have already embarked on developing miniaturized electrochemical biosensors using SPEs, the developed electrochemical aptasensors for the detection of *Salmonella* were found to be mainly fabricated using conventional bulky electrodes (13 studies). Only two studies conducted by Bagheryan et al. [44] and Pathania et al. [45] fabricated electrochemical aptasensors using screen-printed carbon electrodes (SPCE) to detect *S*. Typhimurium.

### 3.4. Detection Method

**Figure 4:**
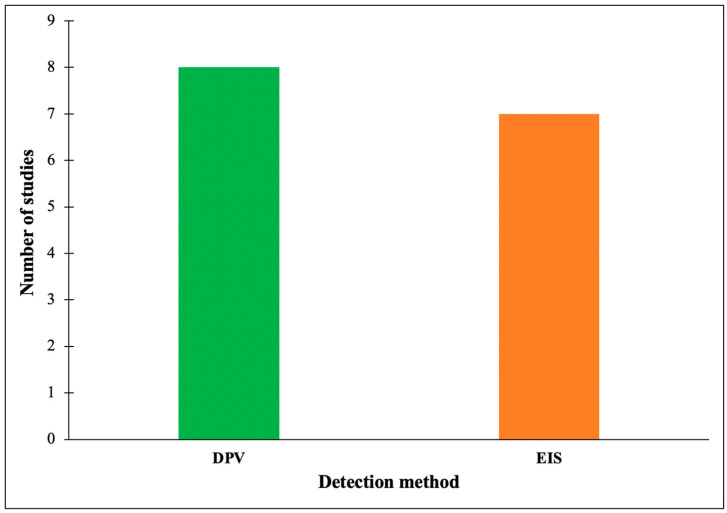
Detection method commonly applied for fabrication of electrochemical aptasensor.

The detection method is one of the critical components in the fabrication of a highly sensitive electrochemical aptasensor platform for the detection of *Salmonella*. Similar to sensor specification, this factor will determine the LoD of the developed electrochemical aptasensor. As shown in Figure 4, the developed electrochemical aptasensors mainly employed differential pulse voltammetry (DPV) (eight studies) and an impedimetric method known as electrochemical impedance spectroscopy (EIS) (seven studies) for electrochemical detection of *Salmonella*.

### 3.5. Assessment of Study Outcomes

The sensitivity of the developed electrochemical aptasensors was evaluated using LoD. All studies presented the LoD in CFU/mL. Of the three aptasensors developed for *Salmonella* spp., the LoD ranged from 3 CFU/mL to 25 CFU/mL. The most sensitive aptasensor utilized a GCE modified with graphene oxide and EIS as the electrochemical detection method [39]. The aptasensor detected *Salmonella* spp. in pork samples within 35 min of incubation time.

With regards to aptasensors developed for *S.* Typhimurium, the LoD ranged from 1 CFU/mL to 16 CFU/mL. The detection time of the assays was between 5 and 180 min. Two studies did not report the detection time of their aptasensors [42,46]. The most sensitive aptasensor utilized a GCE modified with Au and NPG, with EIS as the electrochemical detection method [48]. The aptasensor showed a detection limit of 1 CFU/mL in egg samples with a detection time of 40 min.

For *S.* Enteritidis, an evaluation of the aptasensor’s sensitivity using chicken meat showed a LoD of 550 CFU/mL and detection time of 20 min [52]. The electrochemical aptasensor was fabricated using an ITO electrode modified with MWCNT and EIS for electrochemical detection of *Salmonella*. The aptasensor developed for *S. enterica* was specifically fabricated using a GE electrode that was modified with AuNP and utilized DPV to quantitatively detect *S. enterica* in milk samples. The developed electrochemical aptasensor showed a LoD of 1 CFU/mL and detection time of 40 min [53].

## 4. Discussion

Aptamer is used as a biorecognition molecule in the electrochemical sensing platform to improve the quality of the detection process for digitalizing diagnostic applications [56,57]. Owing to its promising features, various electrochemical aptasensors have been developed and met the requirements of a diagnostic device [58]. The reliability of electrochemical aptasensor for detecting various pathogens, including *Salmonella,* has been reported recently. The present scoping review sought to evaluate the performance of the developed electrochemical aptasensors for *Salmonella* detection as an alternative to the existing diagnostic methods.

This review identified a significant gap in the number of studies on electrochemical aptasensors for detecting *Salmonella* in food samples, with only 15 published research articles available in seven years (2014–2021). Of the 15 studies included in the final review, ten studies focused on the detection of *S.* Typhimurium rather than other *Salmonella* serovars. This is probably because among various *Salmonella enterica* serovars, *S.* Typhimurium was identified as one of the predominant serovars that cause foodborne disease outbreaks in many countries [51,59,60]. According to Xiang et al. [4], foodborne disease outbreaks in China have been primarily attributed to this serotype for many years. A similar finding was reported by Muniandy et al. [61], where *S.* Typhimurium was found as the main serovar that causes food poisoning outbreaks in most ASEAN countries, including Japan, Singapore, Malaysia, and Thailand. Moreover, the emergence of multidrug resistance in *S.* Typhimurium has led to difficulty in clinical treatment, increasing the morbidity and mortality rate among infected animals and humans [62,63,64]. Therefore, the early detection of *S.* Typhimurium is crucial to reduce the risk of death and limit the spread of resistance genes in contaminated food chains. Besides *S.* Typhimurium, two other serovars, *S.* Typhi and *S*. Paratyphi, have also been recognized as the most prevalent serovars that cause foodborne disease among humans resulting in the serious febrile illness known as typhoid fever. These *Salmonella* serovars can only infect humans and may cause death if left untreated [58,65]. However, none of the electrochemical aptasensors were developed to detect these serovars. Therefore, future study needs to focus on developing electrochemical aptasensors for detecting these serovars to reduce outbreaks of the disease.

As shown in Table 2, all 15 studies revealed *Salmonella* detection using the whole cell as the target in food samples, namely, chicken meat (six studies), milk (four studies), apple juice (two studies), egg (two studies), and pork (one study). These are the commonly reported food sources of *Salmonella* [6]. Therefore, the early detection of *Salmonella* in foods is crucial to controlling the outbreak of foodborne disease. Among the various types of food samples, most studies used chicken meat to detect *Salmonella* because poultry products, including chicken meat, possess the highest possibility for *Salmonella* food contamination [54,66]. In the United States, the estimated cost of controlling *Salmonella* infection from poultry products is approximately $11,588 billion dollars a year [67]. Moreover, *S.* Typhimurium is often detected in contaminated poultry, beef, and pork products [68]. The possibility of contamination with *Salmonella* in poultry products occurs during the production, processing, distribution, retail marketing, preparation, and handling processes [66].

In this scoping review, the performance of developed electrochemical aptasensors for detection of *Salmonella* was evaluated based on LoD. The developed electrochemical aptasensors for *Salmonella* detection exhibited LoD ranging from 550 CFU/mL to as low as 1 CFU/mL within 5 min until to 240 min of detection time [41,50,52]. This finding demonstrated that the detection performance of the developed electrochemical aptasensors is comparable to other commercial diagnostic methods for *Salmonella* detection [65]. According to the European Commission (EC) regulation No 2073/2005 on the microbiological analysis of food products, the presence of *Salmonella* at an extremely low concentration, 1 CFU/mL, in ready-to-eat-food (a portion of 25 g) is sufficient to give an infection to humans [69]. Based on the 15 studies analyzed in this review, only two studies reported a LoD of 1 CFU/mL. Therefore, developing highly sensitive electrochemical aptasensors for *Salmonella* detection is essential. Electrochemical detection sensitivity is often determined by several factors, such as the working electrode materials (sensor specification) and electrochemical detection methods [70]. These factors are discussed in detail in this review.

In an electrochemical biosensor, the working electrode serves as the central region for biochemical reactions between the immobilized biorecognition molecules and the target analyte. It must have conductive solid support for the immobilization of biorecognition molecules (aptamer) and electron transports [71]. Accordingly, the electrode materials, their surface modification, and their geometry can significantly determine the sensing performance of the electrochemical aptasensor. Different sensor specifications among the 15 studies has been addressed in this review. Generally, most studies employed GCE (seven studies) and Au (four studies) as the working electrode in the fabrication of electrochemical aptasensors to detect *Salmonella*. This is probably because carbon and Au offer higher electrochemical stability over a wider range of potentials, good biocompatibility with biorecognition molecules, and lower background noise than other metals [72,73]. In particular, Au is most useful in fabricating effective biosensors due to its high electrical conductivity properties, which enable fast electron transfer between redox electrolytes and electrode surfaces. Meanwhile, carbon has been widely utilized in broad electrochemical sensing platforms since it is cheap and compatible with various nanomaterials compared to other noble metals [73,74].

Adding nanomaterials (carbon-based and non-carbon-based) onto the surface of the working electrode will enhance the electrochemical aptasensor characteristics, including the surface area for the immobilization of aptamer, electron transfer kinetics, and electrical conductivity [57,65]. It can be noticed from Table 3 that most studies employed AuNPs (six studies) as nanomaterials in the fabrication of electrochemical aptasensors for the detection of *Salmonella.* This finding was in agreement with other literature that reported AuNPs as the most standing nanomaterials utilized for modifying working electrodes in numerous types of electrochemical biosensors [75,76]. AuNPs exhibit excellent characteristics, including a high surface-to-volume ratio and surface energy due to their nanoscale size, and enhance the electron movement between redox species and the electrode surface [77,78,79]. Moreover, AuNPs have versatility in conjugation with various biomolecules without affecting their biochemical characteristics [80]. These superior characteristics make the developed aptasensor able to detect a LoD as low as 1 CFU/mL and the fastest detection time is within 40 min.

Besides AuNPs, NPG has also been utilized to fabricate electrochemical aptasensors for *Salmonella* detection. NPG is a three-dimensional (3D) nanoporous bulk material synthesized by selective corrosion of Ag from Ag–Au alloys [81]. In addition to good biocompatibility, the 3D porous structure of NPG showed better electrochemical signals and lower detection limits [82]. This could be explained by the fact that the electrochemical aptasensor that was fabricated with NPG exhibited a comparable detection performance to the AuNPs-based electrochemical aptasensor, where the lowest LoD was 1 CFU/mL with a detection time of 40 min [48].

Carbon-based nanomaterials, such as graphene (one study) and its derived nanomaterials, GO (one study) and rGO (four studies), are also employed in constructing electrochemical aptasensors for *Salmonella* detection. Graphene is known as a good surface modifier in electrochemical sensing platforms as it has greater compatibility with diverse biomolecules and microorganisms [83]. In addition, it demonstrates excellent physicochemical characteristics, including large surface area, high thermal conductivity, good electron transfer ability, and mechanical stability [84]. The lowest LoD that the graphene-based electrochemical aptasensor could detect was 3 CFU/mL, with the fastest detection time being 5 min. Despite its excellent characteristics, graphene has a limitation when exposed to a hydrophilic solution due to its hydrophobicity. This limitation can be overcome by the functionalization of graphene with hydrophilic functional groups, namely, carboxyl groups (-COOH) or hydroxyl groups (-OH), to produce a graphite structure called graphene oxide (GO) [71]. rGO was obtained by removing the oxygen-rich functional group on GO through heating or chemical treatment [85]. Although the surface and the edge structure of graphene were modified, the good physicochemical characteristics of both GO and rGO were unaffected [86]. Therefore, these nanomaterials are often chosen as a surface modifier in electrochemical aptasensor development compared to other carbon materials.

The present review also demonstrates the use of MWCNTs (two studies) as the surface modifier for the developed electrochemical aptasensors. Similar to graphene, this is a carbon-based nanomaterial that provides a larger surface area for the immobilization of aptamer and excellent electrical conductivity that allows efficient electron transfer kinetics on the surface of the working electrode [87]. Compared with other nanomaterials, the fabricated electrochemical biosensor using this type of nanomaterial exhibited a high LoD of 550–25 CFU/mL with a detection time range from 20 to 60 min. This could be explained by the limited number of studies utilizing MWCNTs to construct electrochemical aptasensors for *Salmonella* detection.

Generally, the conventional electrochemical method setup consists of three separate electrodes, namely a working, a counter, and a reference electrode, that are immersed into the electrolyte solution and connected to the potentiostat (to control the electric parameters and measure the electrochemical reactions) and a computer (to display the data) [88]. The bulky size of conventional electrodes requires a higher sample volume and reagent for the detection process. The increased demands of the electrochemical biosensor in point-of-care (POC) analysis, particularly in clinical diagnostic applications, urge the transition of conventional bulky electrochemical cell systems to miniaturized electrodes, such as screen-printed electrodes (SPEs) [55,89]. The present review discovered a limitation of the developed electrochemical aptasensors for *Salmonella* detection, namely, that they are mostly constructed using conventional electrodes (13 studies) rather than SPEs (two studies). This finding demonstrates the need for more studies using SPEs to detect *Salmonella* as it offers flexibility in electrode design, material compatibility and modifications, low production cost, the possibility of large-scale production, and connection to portable instrumentation [90,91,92]. Due to their miniaturized size, SPEs can significantly improve detection systems in diagnostic applications since the sample volume and reagent can be reduced to microliters, and low-power analysis is needed. The miniaturized size, versatility, and portability make the SPEs highly possible for on-site testing and monitoring of real samples [93,94]. Unlike conventional electrodes that require a polishing and cleaning process before use, SPEs do not require a tedious cleaning process, which subsequently shortens the detection process [94]. Following the great importance of SPEs in electrochemical sensing platforms, more research needs to be conducted on *Salmonella* detection to improve the quality assessment of this infectious disease.

Another critical component in the fabrication of an electrochemical aptasensor is the electrochemical detection method. The common detection methods utilized to monitor the presence of *Salmonella* in food samples were DPV (eight studies) and EIS (seven studies). These are the most reported electrochemical detection methods that apply to many other infectious pathogens [95]. The voltammetric method is often chosen in most electrochemical diagnostic sensing platforms because the technique is simple and requires inexpensive instrumentation (only a potentiostat is needed) [96]. In the voltammetric method, a specific range of electrical potential is supplied to the working electrode (the region of the target analyte bind), and the resulting current is measured. The current response corresponds to the concentration of the target analyte [97]. As shown in Figure 4, the voltammetric method, namely DPV, was found as the primary detection method for *Salmonella,* with the LoD range from 20 CFU/mL to 1 CFU/mL. The DPV method is beneficial for detecting *Salmonella* at very low concentrations since it possesses a high resolution and sensitivity [98]. In addition to the voltammetric method, the impedance method, namely electrochemical impedance spectroscopy (EIS), has been used to detect *Salmonella.* In the EIS method, the current response on the surface of the working electrode is measured by imposing a small sinusoidal potential (typically ranging from 5 mV to 10 mV), and the changes in frequency (*f*) from the applied potential over a broad frequency range is recorded [99]. In this study, EIS showed a comparable detection performance with the DPV method for *Salmonella* diagnosis. Unlike DPV, the EIS method could give detailed information on the mass-transfer, charge-transfer, and diffusion processes between the electrode, biorecognition molecule, target analyte, and electrolyte. However, understanding EIS theory and its data interpretation are very complicated for those with no background in analytical chemistry (such as biologists, material scientists, or biochemists) [100]. These factors limit the utilization of EIS in the electrochemical detection of *Salmonella*, regardless of its excellent performance.

## 5. Conclusions

This review assessed the potential of electrochemical aptasensors to detect *Salmonella*. The current progress of the electrochemical sensing platform was evaluated through several components, namely, serovar involvement, sample matrix, sensor specification and platform technologies for fabrication, electrochemical detection method, LoD, and detection time. The review revealed that the electrochemical aptasensors were mainly developed to detect *S.* Typhimurium. It is essential to develop electrochemical aptasensors for other serovars, such as *S.* Typhi and *S.* Paratyphi, as these pathogens have also been identified as serious pathogen associated with the outbreak of foodborne disease. The developed electrochemical aptasensors primarily detect the whole cell of *Salmonella* in chicken meat. Through this review, we find that the sensor specification and detection methods influenced the detection performance (LoD and detection time) of the developed electrochemical aptasensors. The most sensitive sensor specifications are GCE and GE, modified with NPG and AuNP, respectively. The following sensor specifications are highly sensitive with DPV and EIS detection methods. These findings suggest that both detection methods can be used for better performance of the electrochemical aptasensors. Intensive research on developing electrochemical aptasensors using SPEs is needed to enable aptamer-based biosensor applications for on-site, portable, and real-time analysis for *Salmonella* detection in food and human samples.

## Figures and Tables

**Table 1:** Characteristics of included studies.

*Salmonella* Serovar	Year of Study	Target	Sample	Sensor Specification	Platform Technology for Fabrication	Detection Method	Limit Of Detection	Detection Time (min)	Reference
*Salmonella* spp.	2014	Whole-cell	Pork	GCE/GO/Aptamer/Cell	Conventional three-electrode system	EIS	3 CFU/mL	35	[39]
	2016	Whole-cell	Chicken meat	GCE/rGO-MWCNTs/Aptamer/Cell	Conventional three-electrode system	EIS	25 CFU/mL	60	[40]
	2016	Whole-cell	Bacterial culture	Au/ssDNA probe/Aptamer	Conventional three-electrode system	DPV	20 CFU/mL	240	[41]
*Salmonella* Typhimurium	2016	Whole-cell	Milk	Au/AuNP/Aptamer /ssDNA	Conventional three-electrode system	DPV	3 CFU/mL	NR	[42]
	2016	Whole-cell	Apple juice	Au/Poly [pyrrole-co-3-carboxyl-pyrrole] copolymer/Aptamer/Cell	Conventional three-electrode system	EIS	3 CFU/mL	45	[43]
	2016	Whole-cell	Apple juice	SPCE/Zn-mediated grafting/Aptamer/Cell	SPE	EIS	6 CFU/mL	40	[44]
	2017	Whole-cell	Egg	SPCE-AuNPs/Aptamer/Cell	SPE	EIS	10 CFU/mL^.^	40	[45]
	2017	Whole-cell	Chicken meat	GCE/rGO/Aptamer/Cell	Conventional three-electrode system	DPV	10 CFU/mL	NR	[46]
	2018	Whole-cell	Milk	Au/AuNP/Aptamer/Cell	Conventional three-electrode system	DPV	16 CFU/mL	60	[47]
	2018	Whole-cell	Egg	GCE/Au/NPG/Aptamer/Cell	Conventional three-electrode system	EIS	1 CFU/mL	40	[48]
	2019	Whole-cell	Milk	GCE/Graphene/AuNP/Aptamer/Cell	Conventional three-electrode system	DPV	5 CFU/mL	180	[49]
	2019	Whole-cell	Chicken meat	GCE/rGO/Aptamer/Cell	Conventional three-electrode system	DPV	10 CFU/mL	5	[50]
	2020	Whole-cell	Chicken meat	GCE/rGO/Aptamer/Cell	Conventional three-electrode system	DPV	10 CFU/mL	10	[51]
*Salmonella* Typhimurium and *Salmonella* Enteritidis	2018	Whole-cell	Chicken meat	ITO/MWCNT/Aptamer/Cell	Conventional three-electrode system	EIS	550 CFU/mL for *S*. Enteritidis 670 CFU/mL for *S*. Typhimurium	20	[52]
*Salmonella enterica*	2021	Whole-cell	Milk	GE/AuNP/Aptamer/Cell	Conventional three-electrode system	DPV	1 CFU/mL	40	[53]

Au = gold; AuNP = gold nanoparticles; GE = graphite electrode; GCE = glassy carbon electrode; GO = graphene oxide; ITO = indium tin oxide; MWCNTs = multi-walled carbon nanotubes; NPG = nanoporous gold; NR = not reported; rGO = reduced graphene oxide; SPE = screen-printed electrode; SPCE = screen-printed carbon electrode; Zn = zinc.

**Table 2:** Sample matrix for detection of *Salmonella* using electrochemical aptasensor platform.

Sample	Number of Studies
Chicken meat	6
Milk	4
Egg	2
Apple juice	2
Pork	1

**Table 3:** Type of nanomaterials utilized as a surface modifier of the working electrode.

Type of Nanomaterials	Number of Studies
Gold nanoparticles (AuNPs)	6
Nanoporous gold (NPG)	1
Graphene	1
Graphene oxide (GO)	1
reduced Graphene oxide (rGO)	4
Multi-walled carbon nanotubes (MWCNTs)	2

## Data Availability

Not applicable.
